# Periodontitis increases rheumatic factor serum levels and citrullinated proteins in gingival tissues and alter cytokine balance in arthritic rats

**DOI:** 10.1371/journal.pone.0174442

**Published:** 2017-03-30

**Authors:** Mônica G. Corrêa, Silvana B. Sacchetti, Fernanda Vieira Ribeiro, Suzana Peres Pimentel, Renato Corrêa Viana Casarin, Fabiano Ribeiro Cirano, Marcio Z. Casati

**Affiliations:** 1 Dental Research Division, School of Dentistry, Paulista University, São Paulo, São Paulo, Brazil; 2 Pediatric Rheumatology Unit, Pediatric Rheumatology Unit, Santa Casa de São Paulo, São Paulo, SP, Brazil; New York Medical College, UNITED STATES

## Abstract

This study investigated some immunological features by experimental periodontitis (EP) and rheumatoid arthritis (RA) disease interact in destructive processes in arthritic rats. Rats were assigned to the following groups: EP +RA; RA; EP; and Negative Control. RA was induced by immunizations with type-II collagen and a local immunization with Complete Freund’s adjuvant in the paw. Periodontitis was induced by ligating the right first molars. The serum level of rheumatoid factor (RF) and anti-citrullinated protein antibody (ACCPA) were measured before the induction of EP (T1) and at 28 days after (T2) by ELISA assay. ACCPA levels were also measured in the gingival tissue at T2. The specimens were processed for morphometric analysis of bone loss, and the gingival tissue surrounding the first molar was collected for the quantification of interleukin IL-1β, IL-4, IL-6, IL-17 and TNF-α using a Luminex/MAGpix assay. Paw edema was analyzed using a plethysmometer. Periodontitis increased the RF and ACCPA levels in the serum and in the gingival tissue, respectively. Besides, the level of paw swelling was increased by EP and remained in progress until the end of the experiment, when EP was associated with RA. Greater values of IL-17 were observed only when RA was present, in spite of PE. It can be concluded that periodontitis increases rheumatic factor serum levels and citrullinated proteins level in gingival tissues and alter cytokine balance in arthritic rats; at the same time, arthritis increases periodontal destruction, confirming the bidirectional interaction between diseases.

## Introduction

Both rheumatoid arthritis (RA) and periodontitis are chronic inflammatory diseases that lead to tissue destruction. Rheumatoid arthritis is a chronic inflammatory polyarthritis with a prevalence of 0.5% to 1.0% in adults in industrialized countries [1]. The etiology is multifactorial and the pathogenesis is not fully understood. One possibility is autoimmunity to citrullinated proteins, which is highly specific for RA and may be related to the pathogenesis of the disease [2]. Periodontitis, per se, is a bacterial chronic inflammatory condition that leads to the occurrence of supporting tissue destruction and is host-mediated by the local production of immune-inflammatory mediators in response to periodontopathogens [3].

In both RA and periodontitis, inflammatory cell infiltration occurs (T lymphocytes, macrophages and polymorphonuclear), which leads to progressive tissue destruction [3, 4]. Moreover, the maintenance of the inflammatory process is mediated by cytokines in RA and also in periodontitis [3, 5, 6]. Moreover, based on these shared characteristics, there is some evidence of a bidirectional pathway of progression.

An important study of The third National Health and Nutrition Examination Survey (NHANES III), included 4461 patients aged 60 years or older, who had undergone both musculoskeletal and dental examinations and showed that rheumatic subjects were more likely to be edentulous [odds ratio (OR) 2.27, 95% confidence interval (CI) 1.56 3.31] and have periodontitis (OR 1.82, 95% CI 1.04 3.20) compared with non-RA subjects [7]. Some other studies reported poorer periodontal status in patients with rheumatoid arthritis compared to systemically healthy patients. Mercado et al. [8, 9] showed that RA patients had a greater level of tooth loss and higher percentage of pockets than the control group (healthy patients), with no differences in plaque index and bleeding on probing. Furthermore, these authors showed that the number of deep pockets (≥ 6 mm) in RA patients was significantly higher than in controls.

On the other hand, some studies showed periodontitis as a risk factor for the development or increasing severity of RA [10, 11]. There is evidence that the treatment of periodontitis can reduce the severity of arthritis [10, 12, 13] by reducing proinflammatory factors such as CRP-reactive protein and erythrocyte sedimentation. Moreover, *Porphyromonas gingivalis* (Pg), a well-known periodontal pathogen, may be important in the RA development process because of its capacity of citrullinate proteins by releasing peptidylarginine deiminase [14, 15] and has been related to RA worsening in animal studies [16, 17, 18, 19]. Some previous studies indicated that Gram-negative periodontal bacteria, in particular Pg, could be associated with an increase in rheumatic factor (RF) levels, by direct stimulation [20] or an immunological response against them [21].

In recent years, studies have been trying to investigate the ways in which both diseases interact and increase their aggressiveness. At the same time an experimental AR rat model showed the potential increase in gingival levels of gelatinase, collagenase, TNF-a and IL-1β in normoreactive rats [22], other studies showed that existing periodontitis significantly influenced the induction and severity of RA [19]. However, more clarification and basic data are still needed to better assess this bidirectional relationship between AR and PD. Considering the above evidence, this study evaluates the impact of experimental rheumatoid arthritis and experimental periodontitis in rats in regards of seric and local mediators that could contribute to the better understanding of RA-PD link.

## Materials and methods

### Animals

Forty adult male Wistar rats (200–300 g—Butantan Institute, Butantã, São Paulo, Brazil.) were used. The rats were acclimatized for 15 days before use and they were kept in temperature-controlled cages, exposed to a 24-h light–dark cycle of equal time, and had free access to water and food ad libitum (Labina, Purina, Paulínia, São Paulo, Brazil) in the Bioterium of Paulista University. The experimental procedure was approved by the Paulista University Institutional Animal Care and Use Committee (205/13 CEP/ICS/UNIP).

### Experimental design

#### Treatment groups

**Fig 1:**
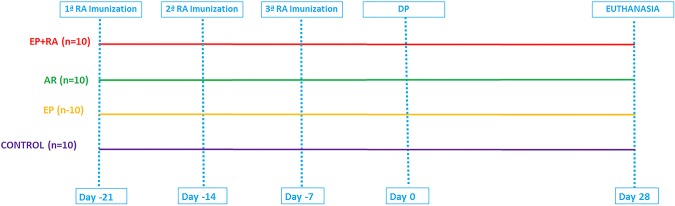
Schematic illustration of the experimental design.

The animals were randomly assigned to one of the following treatment groups: 1- experimental periodontitis + and experimental arthritis (EP+RA) (N = 10); 2- experimental arthritis (RA) (N = 10); 3- experimental periodontitis (EP) (N = 10); 4- Control (n = 10) (Fig 1). This study was carried out in strict accordance with the recommendations in the Guide for the Care and Use of Laboratory Animals of the National Institutes of Health. The experimental procedure was approved by the Paulista University Institutional Animal Care and Use Committee (Permit Number: 205/13 CEP/ICS/UNIP). All surgery was performed general anesthesia by the intramuscular administration of ketamine hydrochloride (0.5 mL/kg; Dopalen, Agribrands, Paulínia, São Paulo, Brazil) and xylazine hydrochloride (10 mg/kg; Rompun, Bayer, São Paulo, São Paulo, Brazil), and all efforts were made to minimize suffering.

#### Rheumatoid arthritis model–RA model

RA was induced by two immunizations of type-II collagen and a third immunization with Complete Freund’s adjuvant (CFA—F5881–10 mL; Sigma-Aldrich, São Paulo, São Paulo, Brazil). The first dose of type-II collagen (C9879-1G - Sigma-Aldrich, São Paulo, São Paulo, Brazil) was administered on day -21 consisting in a total of 0.1 ml emulsion injected at multiple locations at the tail base with a needle (300 μg of type-II collagen in Incomplete Freund’s Adjuvant (IFA—F5506–10 mL; Sigma-Aldrich, São Paulo, São Paulo, Brazil). The second dose, booster dose, was administered subcutaneously on day -14 (injection containing 100 ug of CII in IFA). The second immunization can increase the incidence and severity of arthritis. The third immunization occurred at day -7 through two injections of CFA (1mg/ml) (0.2 ml total): one in the paw subcutaneously (0.1 ml), and the other at the knee joint via intra-articular (0.1 mL). This protocol was based on the studies of Lohman et al. [23] and Sitônio et al. [24], associating with CII/IFA emulsion and CFA immunizations.

#### Rat periodontitis model

To induce experimental periodontitis, one of the mandibular first molars of each animal was randomly assigned to receive a cotton ligature (Corrente Algodão 10, Coats Corrente, São Paulo, São Paulo, Brazil) in a cervical position knotted submarginally. The ligatures were kept in position in order to allow biofilm accumulation over 28 days. The contralateral tooth was left unligated so that it could be used as a control. This procedure was performed under general anesthesia by the intramuscular administration of ketamine hydrochloride (0.5 mL/kg) and xylazine hydrochloride (10 mg/kg).

#### Euthanasia and specimens collecting

Forty-nine days after the start of the study, the animals were euthanized by CO_2_ inhalation. The mandibles were excised for morphometric analysis. The buccal gingival tissue from the area surrounding the lower first molar subjected to experimental periodontitis was also collected for the quantification of immune-inflammatory mediators using the Luminex/MAGpix assay. The collected tissues were placed in sterile tubes containing 400μl phosphate buffered saline (PBS) with 0.05% Tween-20. All samples were stored at -20°C until the imumuno-enzymatic assay. After gingival dissection, the mandibles were de-fleshed after immersion in 8% sodium hypochlorite for 4 h. The specimens were washed in running water and immediately dried with compressed air. To distinguish the cementum enamel junction (CEJ), 1% aqueous methylene blue solution (Sigma-Aldrich, St. Louis, MO) was applied for 1 min to the specimens, which were then washed in running water. Then, the specimens were kept in room temperature into identified cases for posterior photography and morphometry.

#### Measurement of alveolar bone loss

After mandibles preparation as described above, photographs were taken with a 6.1-megapixel digital camera (EOS 40D; Canon, New York, NY, USA) placed on a tripod to keep the camera parallel to the ground at the minimal focal distance. The specimens were fixed in wax with their occlusal planes parallel to the ground and long axes perpendicular to the camera. Photographs of the buccal aspects were taken from both test and control sides. To validate measurement conversions, all specimens were photographed alongside a millimeter ruler [25, 26]. Alveolar bone loss was assessed on the buccal surface of the lower first molars by measuring the distance of the CEJ from the alveolar bone crest at three equally distant sites. The average alveolar bone height of each tooth was calculated. A single examiner, who was unaware of the experimental data, carried out morphometric measurements of alveolar bone loss. The measurements were performed after intraexaminer calibration by evaluating 10 images not taken for this study that show alveolar bone loss similar to the present study. The examiner took the linear measurements of all photographs twice within 24 hours. The intraclass correlation showed 95.7% reproducibility.

#### Rheumatoid factor and anti-citrullinated protein antibody serum levels

Here, 100 μL of blood was collected from the orbital veniplex of rats under anesthesia at T1 (day 0) and at the euthanasia (T2) and the serum samples were stored at −70°C. The level of RF and anti-citrullinated protein antibody in serum aliquots were measured by using the Rat Elisa Kit (MBS720877—monoclonal anti-RF antibody and an RF-HRP conjugate; Mybiosorce, San Diego, California, USA; E-EL-R1431—monoclonal antibody specific to and Avidin-Horseradish Peroxidase (HRP) conjugate -Elabscience, Beijing, China, respectively). The levels of ACCP were also analyzed in gingival tissue. Samples and standards were prepared following the manufacturer’s instructions and analyzed on a fluorescence plate reader.

#### Immunodetection of pro-inflammatory cytokines

To proceed the immune-enzymatic assay the gingival tissue collected was weighed, then cut into small pieces (1 mm^3^ to 2 mm^3^) using scissors and blades [26, 27] and solubilized in PBS to a final concentration of 100 mg tissue/ml. After extraction on a Vortex mixer for 10 min, each sample was centrifuged at 370 g for 5 min, and the supernatant was collected, divided into small portions, and stored at -70°C until use. To avoid protease activity, the entire procedure was carried out at 4°C. The levels of IL-1β, IL-4, IL-6, IL-17 and TNF-α were determined by Luminex/MAGpix assay using commercially available kits (RCYTOMAG-80K; Millipore, Billerica, MA, USA) and following the manufacturers’ instructions. Initially, a 96-well plate was pre-wet with washing buffer, and after discarding the wash buffer, microsphere magnetic beads coated with monoclonal antibodies against the 3 different target analytes were added to the wells. Samples and standards were added to the wells and incubated overnight at 4°C. The wells were washed using a magnetic manifold, and a mixture of biotinylated secondary antibodies was added. After incubation for 1 h, streptavidin conjugated to the fluorescent protein, Rphycoerythrin (streptavidin-RPE), was added to the beads and incubated for 30 min. After washing to remove the unbound reagents, sheath fluid‡‡ was added to the wells, and the beads (minimum of 50 per analyte) were analyzed in the MAGpix™ instrument (MAGpix™ MiraiBio, Alameda, CA, USA). Samples were diluted with the kits’ diluents. The dilution was taken into consideration when calculating the concentration of each substance with a standard curve, prepared using the standard proteins in the kit. The standard curve range used for IL-1β measurement was 2.4–10,000 pg/ml; for IL-4 measurement, 4.9–20,000 pg/ml; for IL-6 7.3–300,000 pg/ml, for TNF-α 2.4 to 10,000 pg/ml; and for IL-17 7.3 to 30,000 pg/Ml.

#### Paw swelling analysis

Systemic macroscopic features of arthritis regarding paw swelling (as measured in ml by plethysmometer, Ugo Basile) [28–31] were recorded every week until the end of the experiment.

#### Statistical analysis

To test the null hypothesis that RA had no influence on alveolar bone loss and cytokine levels, intergroup analysis was performed through a two-way ANOVA test followed by a Tukey test. RF data were analyzed through ANOVA/Bonferroni test. Kruskal-Wallis test was performed for the intergroup analysis of ACCPA and Wilcoxon test for the intragroup comparisons. Plethysmometer values were analyzed by Friedman (intragroup) and Kruskal-Wallis (intergroup) tests. The significance level established for all analyses was 5% [Statistical Analysis System (SAS) 9.3, Cary, NC, USA].

## Result

### Clinical analysis

**Fig 2:**
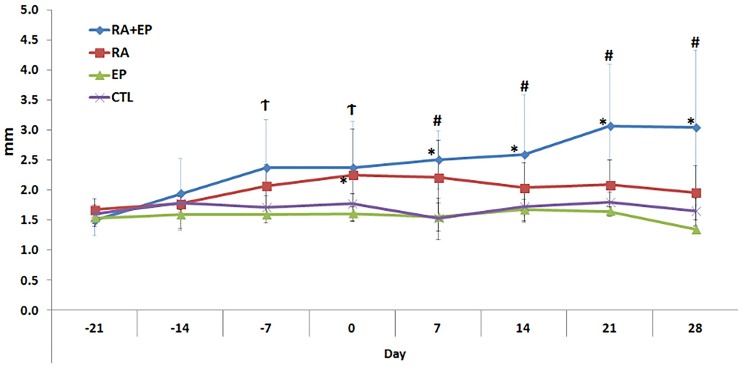
Means a± SD of paw swelling (volume—ml) measured by plethysmometer. * Indicate significant difference to baseline (-21) and † indicate significant difference between RA and EP+RA [Friedman (intragroup) and Kruskal-Wallis (intergroup) p<0.05]. (CONTROL: n = 10; EP: n = 10; RA: n = 10; EP+RA: n = 10).

The animals did not show any signs of systemic illness (except RA) throughout the study period. The rats also did not lose weight throughout the experimental period. No deaths were observed. Twenty-one days after immunization, joint swelling was observed first in the hind paws, and then joint swelling extended to the forelegs and tail. The RA peak occurred on day 28, with multiple and symmetrical joint swelling and redness. Some rats presented deformity and limited mobility in some joints. The greater paw volume was observed especially at the paw that received the CFA injection. Considering the paw swelling assessed by plethysmometer, efficiency in the RA model could be noted once the RA and RA+EP groups presented an increase in swelling after RA induction (days -7, -14, -21 and 28 days), with a significant difference to the control and EP groups at -7 and 0 days (p<0.05). However, after EP induction (day 0), RA groups showed a trend towards a reduction in paw swelling, while RA+EP maintained its volume, being only RA+EP group, in contrast to the control and EP groups until the end of the study (Fig 2 - Figure A in S1 File). Meanwhile, AR symptoms (joints swelling and deformities) lasted until the end of the experimental period in both RA and RA+EP groups. In addition, at the time of euthanasia, clinical examination revealed signs of gingival inflammation, including color/volume changes and bleeding around the ligated teeth of all groups, with no signs of inflammation at the non-ligated sites (contralateral teeth).

### Morphometric results

**Fig 3:**
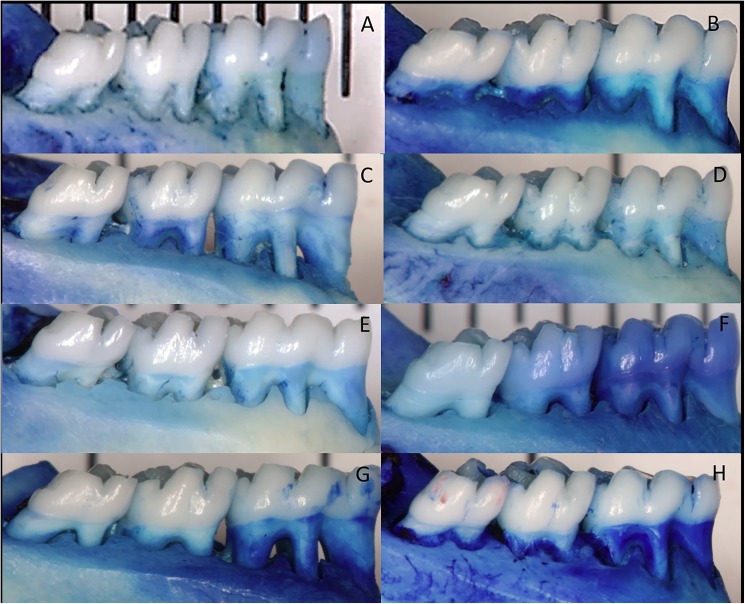
Representative photographs illustrating the morphometric findings of the groups–A–CONTROL (N = 10): Ligated teeth; B–CONTROL (N = 10): Unligated teeth; C–EP (N = 10): Ligated teeth; D–EP (N = 10): Unligated teeth; E–RA (N = 10): Ligated teeth; F–RA (N = 10): Unligated teeth; G–EP+RA (N = 10): Ligated teeth; H—EP+RA (N = 10): Unligated teeth.

Intragroup analysis showed significant differences in alveolar bone loss between unligated and ligated teeth in all groups (P < 0.05). Intergroup analysis of morphometric outcomes showed greater periodontal destruction in the EP+RA group when compared with the control group, PE and RA (p <0.05). The EP group also presented higher bone loss values than the control and RA groups (p<0.05). No difference between groups were noted in unligated teeth (p>0.05). The morphometric findings are shown in Table 1 (Table A in S1 File) and Fig 3 illustrates the morphometric findings.

**Table 1:** Mean ± SD of alveolar bone loss (millimeters) for ligated and unligated teeth.

	MEAN LIGATED±SD	MEAN UNLIGATED±SD	*p-value (intragroup)*
**EP+RA (n = 10)**	1.59±0.09 *	1.28±0.14	*0*.*0001*
**RA (n = 10)**	1.23±0.09 †	1.24±0.14	*0*.*18*
**EP (n = 10)**	1.41±0.07	1.19±0.11	*0*.*026*
**CONTROL (n = 10)**	1.16±0.14 †	1.18±0.13	*0*.*69*
***p-value (inter-group)***	*<0*.*001*	*0*.*184*	

* Significant difference from CONTROL, EP and RA (two-way ANOVA/Tukey test; p<0.05).

† Significant difference from EP (two-way ANOVA/Tukey test; p<0.05).

### RF and ACCPA levels

In T1 (day 0 –after RA induction and prior to EP induction), the levels of RF were higher in EP+RA and RA groups when compared to control and EP groups (p < 0.05) (Table 2 –Table B in S1 File). However, in the EP+RA group, serum levels of RF increase from T1 to T2 (p < 0.05). Thus, at T2 (day 28 after EP and the time of euthanasia) the EP+RA group presented the highest serum concentration of RF (p<0.05), which was also higher than the RA group (p<0.05). Moreover, the RA group still had higher levels of RF compared to the control and EP groups (p < 0.05).

**Table 2:** Means ± SD of rheumatoid factor (RF) serum levels (U/ml) measured by ELISA assay.

	T1	T2	*p-value (intragroup)*
**EP+RA (n = 10)**	77.8 ± 3.8a *	85 ± 2.7a †	*<0*.*0001*
**RA (n = 10)**	76.6 ± 3.1a *	78.1 ± 3.7	*0*.*17*
**EP (n = 10)**	73.5 ± 2.1	72.7 ± 3.4c ‡	*0*.*58*
**CONTROL (n = 10)**	72.4 ± 5.3	71.0 ± 4.9c ‡	*0*.*28*
***p-value (intergroup)***	*0*.*038*	*<0*.*0001*	

* Significant difference from EP and CONTROL in T1 (two-way ANOVA/Tukey test; p<0.05).

† Significant difference from RA, EP and CONTROL in T2 (two-way ANOVA/Tukey test; p<0.05).

‡ Significant difference from RA in T2 (two-way ANOVA/Tukey test; p<0.05).

T1: day of experimental periodontitis induction; T2: euthanasia.

Considering serum levels of ACCPA, the EP+RA and RA groups had higher amounts of anti-citrullinated protein antibody than the control and EP groups in T1 (p < 0.05). Interestingly, ACCPA showed a continuous increase over time in both RA and EP+RA, while no significant changes occurred in the control or EP groups. In T2, the control group presented lower values of ACCPA when compared to EP+RA and RA (p < 0.05). (Table 3 –Table C in S1 File). In gingival tissues, collected at the end of the experimental period (T2), the EP+RA group had the highest value of ACCPA (p<0.05), although the EP group presented higher levels than the RA, but not than control groups (p < 0.05) (Table 3 -Table C in S1 File).

**Table 3:** Means ± SD of anti-citrullinated protein anti-body (ACCPA) serum and gingival levels (U/ml) measured by ELISA assay.

	T1	T2	*p-value(intragroup)*	GINGIVALTISSUE
**EP+RA (n = 10)**	5.7 ± 2.8 a *	19.2 ± 8.1 †	*0*.*0051*	50.8+20.5 ‡
**RA (n = 10)**	9.5 ± 6.5 a *	36.4 ± 26.2 †	*0*.*3329*	0.1+0.1 $
**EP (n = 10)**	0.2 ± 0.2	10.2 ± 11.2	*0*.*0929*	1.1+1.2
**CONTROL (n = 10)**	2.4 ± 0.4	2.3 ± 0.3	*0*.*0680*	0.2+0.1
***p-value (intergroup)***	*<0*.*0001*	*0*.*0002*		*<0*.*0001*

* Significant difference from EP and CONTROL in T1 (Wilcoxon/Kruskal-Wallis test; p<0.05).

† Significant difference from CONTROL in T2 (Wilcoxon/Kruskal-Wallis test; p<0.05).

‡ Significant difference from RA, EP and CONTROL (Newman-Keuls; p<0.05).

$ Significant difference from RA, EP and CONTROL (Newman-Keuls; p<0.05).

T1: day of experimental periodontitis induction; T2: euthanasia.

### Gingival tissue interleukin

Multiplex analysis of inflammatory markers in gingival tissues revealed a higher level of IL-1β in the EP+RA, RA and EP groups than the control one (p<0.05). Considering IL-4 concentration, there was no difference among the groups (p>0.05). The levels of IL-6 and TNF-α were higher in the EP+RA and RA groups, when compared with the control group (p < 0.05). Interestingly, the IL-17 amount was higher in the EP+AR and RA groups when compared to the control and EP groups (p<0.05). (Table 4 - Table D in S1 File).

**Table 4:** Means ± SD of the mean of IL-4, IL-1β, IL-6, IL-17 and TNF-α concentrations (picograms per milliliter) measured by multiplex assay.

	IL-1β	IL-4	IL-6	TNF-α	IL-17
**EP+RA (n = 10)**	1.02+0.39	0.04+0.04	0.64+1.01	0.06+0.04	0.13+0.14
**RA (n = 10)**	1.52+1.07	0.08+0.03	0.42+0.35	0.08+0.09	0.17+0.09
**EP (n = 10)**	1.21+0.24	0.02+0.01	0.21+0.20	0.03+0.01	0.05+0.02b $
**CONTROL (n = 10)**	0.66+0.30b *	0.07+0.08	0.07+0.06b †	0.03+0.02b ‡	0.05+0.03b $
***p-value***	*0*.*048*	*0*.*65*	*0*.*006*	*0*.*03*	*0*.*0002*

* Significant difference from EP+RA, RA, EP and CONTROL (two-way ANOVA/Tukey test; p<0.05).

† Significant difference from EP+RA and RA (two-way ANOVA/Tukey test; p<0.05).

‡ Significant difference from EP+RA and RA (two-way ANOVA/Tukey test; p<0.05).

$ Significant difference from EP+RA and RA (two-way ANOVA/Tukey test; p<0.05).

## Discussion

There is some evidence about the bidirectional pathway to the development of rheumatoid arthritis and periodontitis, although there is no clear pathway by which it occurs. This study evaluated local and systemic markers in arthritic rats submitted to experimental periodontitis. The results show that, in the presence of RA, periodontal breakdown was higher than PE alone, although it was effective in promoting more bone resorption compared to the control and RA groups. Interestingly, although RA and/or EP modulate the local release of cytokines, the RA, per se, seems to increase the local production of IL-17. However, when RA and PE were simultaneously induced, ACCPA and RF were directly affected by the presence of gingival inflammation, increasing their levels, including the local levels of ACCPA. Additionally, the clinical feature of RA, paw swelling, appear to be worsened by periodontitis.

It has been shown that Th17 profile has a critical role in RA and collagen-induced arthritis pathology [32, 33]. IL-17, a proinflammatory cytokine produced by RA synovium [32], stimulates the macrophages’ production of IL-1 and TNF-a [34] and activates human synoviocytes to produce IL-6, IL-8, GM-CSF, and PGE2 [35, 36]. When endogenous IL-17 was blocked, amelioration of collagen-induced arthritis was observed at the same time as its overexpression enhanced synovial inflammation and joint destruction [33]. In the same way, IL-17 is also related to periodontal tissue destruction. Th17-type response triggers osteoclastogenesis, as well as induction of the receptor activator of nuclear factor-kB ligand (RANKL) [37, 38, 39]. In the present study, greater values of IL-17 was observed only when RA was present, in spite of PE, confirming the major role of arthritis in IL-17 production indirectly collaborating to periodontal breakdown. However, the fact that nor IL-17, neither IL-1β, TNF-a, IL-6 or IL-4 were specifically increased/decreased when both pathologies were induced, and the higher periodontal destruction observed at EP+RA, might be related to other inflammatory mechanisms. Queiroz-Junior et al. [40] associated RA and EP in mice and found enhanced expression of the Th1 immune responses associated with worse RA [41] and EP [42] prognosis. Additionally, the same authors observed a reduction in the expression of transcription factors associated with the control of EP (T regulatory cells—Foxp3), and the Th2 response (GATA-3) [5]. This could help to explain the worst periodontal conditions observed in subjects affected by RA observed in clinical studies [8, 9].

On the other hand, some recent findings have indicated that periodontal disease can worsen the severity of collagen-induced arthritis [16, 19, 43]. De Aquino et al. [16] used a bacteria periodontitis inducing model (using mixed *P*. *nigrescens* and *P*. *gingivalis*) and showed an exacerbated arthritis, with increased arthritic bone erosion, by the activation of CII-specific T cell response toward the Th17 phenotype without affecting Th1. The authors found that the levels of IL-17 induced by periodontal pathogens in CII-specific T cells was correlated with the intensity of arthritic bone erosion.

Another point of view is the potential of periodontal pathogens in modulate “destructive” arthritic markers. In the present study, experimental periodontitis was induced in previously arthritic rats (RA was induced 21 days before). Interestingly, greater amount of ACCPA in the serum and gingival tissue of the EP+RA group than in RA or EP alone (and control group, as well) was seen. This phenomenon could be linked to the presence of periodontal-pathogens in subgingival biofilm [44], once the model was described as a plumicrobial infection [45]. Although the present study has used the ligature-induced periodontitis model and has not performed identification analysis, the microbial profile observed by other authors in the ligature model was similar to that observed in human periodontitis [46, 47]. Through a DNA-DNA checkerboard analysis, a previous study identify microbial profile in subgingival environment after ligature-induced periodontitis and found streptococcus- and *Actinomyces*-like species in high mean counts, followed by *Fusobacterium*-, *Prevotella nigrescens*-, *Parvimonas micra*-, *Aggregatibacter actinomycetemcomitans*- and *Porphyromonas gingivalis*-like species [47]. Another study evaluated the presence and concentrations of *Porphyromonas gingivalis*, *Tannerella forsythia* and *Aggregatibacter actinomycetemcomitans* in the cotton ligatures used to induce periodontitis by real-time PCR and it was possible to identify and quantify all the three species in the analyzed samples [46]. This reaffirm the reliability of ligature model to mimic the human periodontal infection. The Red Complex (*Porphyromonas Gingivalis*, *Treponema denticola* and *Tannerella forsythia*) [47] found in the subgingival plaque, is the main group of bacteria related to periodontitis. Among the three species of red complex, Pg has been related to the pathogenesis of RA and may be important in the disease development processes because of its capacity of citrullinate proteins by releasing peptidylarginie deiminase [14, 15]. Recently, *Aggregatibacter actinomycetemcomitans* (Aa) was identified as a potencial trigger of autoimmunity in RA. The study observed induced hypercitrullination in host neutrophils by the pore-forming toxin leukotoxin A (LtxA), reproducing membranolytic pathways that occurs in the RA joint, supporting the autoantigen citrullination [48]. The authors also verified that LtxA induced changes in neutrophil morphology with the release of hypercitrullinated proteins. Additionaly, exposure to leukotoxic Aa strains was confirmed in patients with RA and periodontitis and was associated with both anticitrullinated protein antibodies and rheumatoid factor levels [48]. Interestingly, these authors did not find association of Pg with hipercitrullination in neutrophils in periodontitis samples [48]. Our results also showed that the association of periodontitis and rheumatic arthritis increased the serum levels of citrullinated proteins from T1 (the day of EP induction) to T2 (euthanasia). Interestingly, at the end of the experimental period, the levels of ACCPA in the EP group were similar to the groups with RA. This is in accordance with the results of Konig et al. [48], which demonstrated that LtxA from Aa hypercitrullinated neutrophis in similar process of RA joint. Accordingly, the serum levels of rheumatoid factor increased after the association of EP with experimental RA and the amount of this substance was greater in the EP+RA group when compared to the other groups at the end of the experiment. Thé and Ebersole [49] showed that the RF of seropositive patients show a cross-reaction with oral bacterial epitopes. Moreover, Bonagura et al. [50] verified that Pg proteinase is responsible for the epitope development in the RF-Fc (region of an antibody that interacts with cell surface receptors) region, probably due to the capacity of Pg in decompose lysine and arginine [51, 52], once both aminoacids were found in Fc regions of the IgG molecule [53]. Konig et al. [48] verified significant association of anti-LxtA positivity in RA and ACCPA and RF, when compared to anti-LtxA antibody–negative RA. In this vein, it takes clear that Pg and Aa play an important role in the RF production of rheumatoid cells and their increase in arthritic rats after periodontitis induction.

Meanwhile, the discussion of the possible clinical impact of these findings is important. Rheumatoid factor and the anti-citrullinated protein antibody are highly specific and sensitive markers for rheumatoid arthritis and, importantly, they have been associated with poor outcomes of rheumatoid arthritis, such as increased disease activity, radiographic progression and disability [54, 55, 56]. Besides, ACCPA can be detected very early and predict clinical disease outcome [54, 55, 56]. Considering the fact that paw swelling, a clinical aspect of experimental RA, also showed an impact of periodontal disease, our findings could confirm the negative influence of periodontitis on the development and exacerbation of rheumatoid arthritis; this was show in some previous clinical trials [7, 8, 9] and explains how oral conditions jeopardize arthritic conditions.

In the meantime, the citrullination of protein is a process that can also influence both diseases. Pg-associated citrullination by peptidylarginine deiminase enhance its capacity of surviving in biofilms [50, 51]. On the other hand, citrullinated residues are generated by deamination of the guanidino group of carboxyterminal arginine residues on a variety of peptides, producing citrulline and free ammonia, by a peptidylarginine deiminase. Ammonia interferes with neutrophil function [57, 58] and protects against acidic cleansing cycles of mouth. Moreover, LtxA from Aa changes neutrophil morphology [48] and this may also interferes with its function. Besides, citrullination alters the activity of the complement system, inactivates epidermal growth factors and induces the production of prostaglandin, E2, with all events enrolled in the periodontal destruction [59, 60, 61, 62]. Thus, the increased ACCPA, not only deteriorate arthritic status, but, once it is increased in gingival tissues, could also affect the periodontal destruction. This is the first study to assess locally produced ACCPA and this innovative finding should be evaluated in the future regarding its effect on periodontal breakdown.

In general, our findings confirmed some previously reported conclusions. Rheumatoid arthritis can worsen periodontal destruction, as well as periodontitis altering some arthritic markers, as shown in the analysis of paw swelling. Only the RA groups (RA+EP and RA) presented differences related to the baseline. Differences was observed among groups presenting RA (RA+EP and RA) and other groups without RA (EP and Control). Interestingly, when EP was associated with RA, the paw swelling remained in progress until the end of the experiment, with a significant difference when compared to EP and control. This result is in agreement with Cantley’s study [19], where the association of arthritis and periodontitis resulted in higher paw swelling over the time when compared to RA alone. Besides, some new findings can highlight and strengthen this bidirectional relationship. ACCPA level increase in gingival tissues also should be considered to explain this interaction, as well as the role of TH17 pathway on both diseases. The evident impact of periodontitis in rheumatoid factor levels also brings new light to this. All of these findings, based on shared patterns, could reinforce the idea of a bidirectional relationship between them.

Thus, with the limits of this study, it can be concluded that rheumatoid arthritis enhances periodontal tissue destruction. RA alone does not enhance bone loss, but increases the concentration of inflammatory cytokines, especially IL-17. Periodontitis increases the serum levels of rheumatoid factor, as well as the gingival tissue concentration of ACCPA, confirming the bidirectional aspect of both diseases.

## Supporting information

S1 File Supporting Information underling the findings. (XLSX)
